# Environment or Pollinators? Factors Shaping Breeding System and Spatial Variation in Nectar Properties and Pollination System in a Desert Species *Fritillaria persica* L. (Liliaceae)

**DOI:** 10.1002/ece3.71265

**Published:** 2025-04-25

**Authors:** Katarzyna Roguz, Paweł Pstrokoński, Justyna Ryniewicz, Magdalena Chmur, Andrzej Bajguz, Yuval Sapir

**Affiliations:** ^1^ Botanic Garden Faculty of Biology, University of Warsaw Warsaw Poland; ^2^ Department of Animal Breeding Institute of Animal Sciences, Warsaw University of Life Sciences Warsaw Poland; ^3^ Department of Plant Biochemistry and Toxicology Institute of Biology, Faculty of Biology and Chemistry, University of Bialystok Bialystok Poland; ^4^ Yehuda Naftali Botanic Garden School of Plant Sciences and Food Security, Faculty of Life Science, Tel Aviv University Tel Aviv Israel

## Abstract

Interaction with pollinators has been proposed as one of the most important factors shaping the diversity of flowering plants. Spatial variation in the directions of the selective pressure exerted by pollinators drives the evolution of adaptive differentiation. Across‐population studies of flower traits and plant–pollinator interaction are therefore an important step to understanding the diverse selective pressures that drive floral evolution in zoogamous angiosperms. Here we combine observational data and field experiments to describe the assemblages of pollinators, breeding systems, and reward properties in studied populations of the Middle East geophyte, *Fritillaria persica*. Natural populations of this species include two floral color morphs with greenish or purple flowers; in both morphs, the nectaries of the outer whorl are covered by the tepals of the inner one. Our study documented geographical variation in the pollination system of two color morphs of *F. persica* . Visitors recorded in both populations were similar qualitatively; however, their contribution varied. Nectar sugar concentration and profile were generally constant in studied populations; we recorded differences only in nectar volume and concentration of amino acids. These results suggest that the observed variation in nectar production is likely to be a result of environmental factors rather than pollinator‐mediated selection. In the context of reward, we also tested how uncovering the hidden nectar reward from outer tepals influences potential pollinators. Uncovering hidden reward did not change the time spent in one flower or inflorescence penetration; however, it increased the number of seeds produced. Nectar properties and the pollinator assemblages similarity suggest that in the context of pollination, *F. persica* represents a rather generalistic strategy, and observed differences may be caused by abiotic factors.

## Graphical abstract

Our study documented geographical variation in the pollination system of two color morphs of Fritillaria persica, a desert geophyte. Visitors recorded in both populations were similar qualitatively, and nectar sugar concentration and profile were generally constant in studied populations; we recorded differences only in nectar volume and concentration of amino acids. These results suggest that the observed variation in nectar production is likely to be a result of environmental factors, rather than pollinator‐mediated selection.

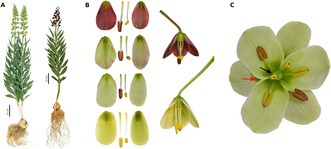

## 1 Introduction

Pollination by animals is considered the most ecologically important interaction between plants and animals, as it is often crucial for plant reproductive success. The reproduction of 87.5% of all wild plant species depends on animal pollination (Ollerton et al. 2011). Typically, plants provide pollinators with a food reward of either pollen or nectar. Among these, nectar is the primary reward offered to pollinators in the majority of angiosperms and is perhaps the most important in an evolutionary context, playing a key role in shaping the relationship with pollinators (Brzosko et al. 2021; Canto et al. 2011; Parachnowitsch et al. 2018; Roguz et al. 2018, 2021; Roy et al. 2017; Simpson and Neff 1981). The amount, composition, and accessibility of nectar are central to pollination ecology, as they influence the behavior and preferences of pollinators and ultimately impact plant reproductive success (Palmer‐Young et al. 2019; Parachnowitsch et al. 2018; Willmer 2011). Nectar properties contribute to the learning of the pollinators if the signal is honest, that is, a positive correlation exists between the amount and concentration and the floral signal. Hence, nectar traits are thought to be under pollinator‐mediated selection (Eisen et al. 2023; Ortiz et al. 2021; Parachnowitsch et al. 2018; Schaefer et al. 2004).

Functionally, nectar is a nutrient‐rich aqueous solution containing mostly sugars, as well as low concentrations of amino acids (AAs), proteins, fats, organic acids, and other minor components such as minerals, vitamins, and oils (Nicolson and Thornburg 2007). Its sugar composition can vary greatly depending on the plant species (Herrera et al. 2006), being often a mixture of glucose, fructose, and sucrose in varying proportions, with sucrose being the main component (Lotz and Schondube 2006). Sugar concentration differences have implications for plant–pollinator interactions, as they could be linked to pollinator preferences for specific properties, challenges associated with handling viscous/solid nectars, and plant energy allocation strategies aimed at minimizing the cost of nectar production (Lanza et al. 1995). Moreover, sugar concentration plays a major role in the immediate reaction of plants to their pollinators, as the sugar amount in the nectar can increase in minutes (Veits et al. 2019).

After sugars, AAs are the second most abundant constituents in floral nectar. The presence of AAs and other non‐sugar constituents enriches floral nectar with nutritional values beyond just a source of simple chemical energy (Nepi 2014). AAs are typically found in nectar at low but measurable quantities (0.02%–4.8% organic matter), and they have been proposed as one of the most important features shaping plant–pollinator interactions (Fornoff et al. 2017). Floral nectar serves as a source of essential amino acids (EAAs), which are crucial for growth, somatic maintenance, and reproduction (Mevi‐Schütz and Erhardt 2005). Additionally, the oxidative proline degradation pathway utilizes proline as a source of energy, specifically during the initial phase of insect flight (Carter et al. 2006; Teulier et al. 2016).

Nectar concentration and composition are highly variable traits and pose the most intriguing questions concerning the adaptive evolution of flower traits. It is still debated whether the presence and proportion of nectar components are adaptive in relation to pollinator type, and whether nectar composition is dictated by phylogeny, or is a secondary consequence of flower morphology and environmental factors (Willmer 2011). Nectar concentration and composition can vary within the flower and inflorescence, between individuals, and among populations. Interspecific differences in nectar volume, sugar, and AAs concentration and composition have often been interpreted as adaptations to pollinator preferences (Parachnowitsch et al. 2018; Willmer 2011).

Stebbins' theory suggests that the selection on floral traits is driven by the most efficient pollinator, implying that the adaptation of floral traits can exhibit pollinator specificity (Stebbins 1970). This selection may be mirrored by nectar properties (Dellinger et al. 2019, 2021; Shrestha et al. 2019; Sletvold et al. 2016). Moreover, pollinator assemblages interacting with specific plant communities can vary in species composition or proportion (quantity component; Gómez et al. 2009; Gómez and Zamora 2006; Zhao and Huang 2013). Additionally, the behavior and effectiveness of pollinators can differ among populations (quality component; Hersch and Roy 2007; Zych et al. 2019).

The selection on floral traits shaping interaction with pollinators is expected to be stronger in specialized pollination systems compared to generalized ones (Aigner 2001; Johnson and Steiner 2000). Nonetheless, even in generalist species, there can be pollination‐related changes in flower traits leading to “cryptic specialization” (Bell 1971), usually expressed in a mosaic of floral traits related to the pollinator community (Thompson 1999). In some generalist species, pollinated by disparate pollinator functional groups, there is little evidence for distinct pollination ecotypes, and the observed variation in floral features is interpreted as “adaptive wandering” (Zych et al. 2019). In this process, different populations of the same species may diverge in response to local pollinator communities. It does not result in a pollinator shift because the direction and strength of selective pressure act too briefly to cause any substantial morphological and phenotypic changes (Wilson and Thomson 1991).

Plants can be plastic in their nectar production, responding to a range of environmental factors such as drought (Waser and Price 2016), ambient humidity and temperature (Bertsch 1983; Petanidou et al. 2006; Petanidou and Smets 1996), soil properties (Gardener and Gillman 2001; Ryniewicz et al. 2020), elevated CO_2_ (Dag and Eisikowitch 2000), interactions with herbivores (Aizen and Raffaele 1996) or nectar microbes (Vannette 2020). Recorded changes in nectar properties caused by surrounding microclimate indicate that nectar can be particularly sensitive and show changes in phenotypic measurements. However, there are also studies suggesting that in some species, nectar properties may be less influenced by environmental factors (Lehtilä and Strauss 1997).

**FIGURE 1:**
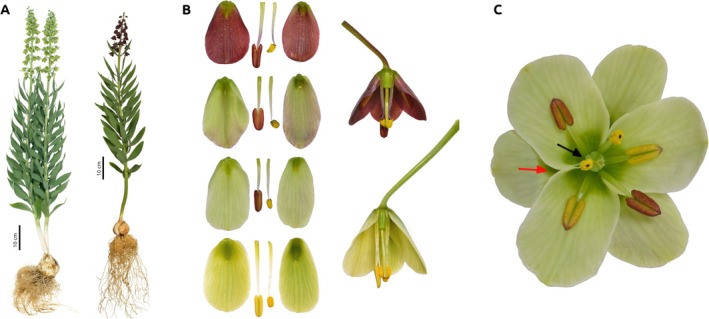
(A) Studied species *Fritillaria persica* in two color morphs. (B) The continuous coloration of *F. persica* flowers found in natural populations. (C) The movement of reproductive parts within flowers. At the beginning of the flowering, the unripe stamens lay flat on the tepals at a distance from the style. The stamens position changes as the flower grows—the male parts gradually shift towards the center and finally, they come into contact with the stigma, depositing pollen on it. Also (C) The nectaries of inner whorl visible (black arrow) and outer whorl (red arrow) covered by the tepals of inner whorl.

The variety of possible selection mosaics occurring in the context of spatial variation indicates the need for further studies if we aim to understand the diversity of flowering plants. Interspecific variation in floral traits, nectar characteristics, and plant–pollinator interactions are important steps in understanding the diverse selective pressures that drive floral evolution in zoogamous angiosperms (Aigner 2004; Chapurlat et al. 2015; Herrera et al. 2006). Here, we test the possible association between intraspecific variation in reward properties and variation in pollination systems of the Persian lily, *Fritillaria persica* L. (Liliaceae). This is an ornamental bulbous plant, which belongs to the monotypic subgenus *Theresia* Koch of the genus *Fritillaria* (Day et al. 2014). The species is native to the East Mediterranean and Western Asia (Iran, Iraq, Turkey, Cyprus, Syria, Israel, Palestine, and Jordan) with unusually large inflorescences, often containing more than a dozen flowers (Tekşen and Aytaç 2011). While in most populations across the Middle East, the flowers are almost monomorphic, cream, or greenish‐cream, some populations exhibit color polymorphism, with flower colors varying from dark or pale purple to greenish‐purple, white, or rarely creamy, on a continuous color scale (Figure 1A,B). *Fritillaria persica* has a distinctive arrangement of reproductive organs; it is the only known *Fritillaria* species in which part of the nectar reward is hidden—the nectaries of the outer tepals are shielded by the inner tepals and are likely not easily accessible to visiting insects (Roguz et al. 2018; Figure 1C). Currently, the reproductive biology of *F. persica* is virtually unknown and, to date, plant–pollinator interactions in this species in natural populations have never been studied. The existence of color polymorphism and populations located at varying spatial distances make *Fritillaria persica* an excellent system to study spatial variation in flower features and pollination systems. Moreover, this species has a distinctive arrangement of reproductive parts when compared to other representatives of the genus. Available data suggest that the species is pollinated by bees (Roguz et al. 2018), and based on the reward properties and arrangement of reproductive parts, most likely represents a semi‐generalized model of pollination. In this study, we explored the breeding system, nectar properties, and the assemblages of pollinators in populations of *F. persica* . We experimentally manipulated the accessibility of pollinators to the nectar to assess the role played by limited access to the reward. We aimed to answer the following questions: (1) Are there differences in reward properties among populations with different flower colors? (2) If yes—how do these differences in nectar characteristics influence the interaction with pollinators and contribute to the specialized pollination system of *F. persica* ? (3) What is the role of the hidden nectar in the interaction with pollinators?

## 2 Materials and Methods

### 2.1 Study System

*Fritillaria persica* L. produces bell‐shaped flowers, borne in dense clusters on pyramidal racemes along leafy stems. Each raceme produces 10–50 small flowers, with a perigon length of 10–20 mm. *Fritillaria persica* is morphologically androdioecious, with flowering specimens having racemes composed only of flowers of the same sexual morph, either hermaphrodite or male (Mancuso and Peruzzi 2010). Flowers open successively on the stem and are typically open for a few days. Stigmas are receptive throughout flowering, and pollen is available for a few days, depending on the activity of the visitors, until the anthers dehisce (Mancuso and Peruzzi 2010).

*Fritillaria persica* has a distinctive arrangement of reproductive organs. The position of the stamens changes throughout the flower's lifetime, starting in a peripheral position almost attached to the petals, gradually shifting towards the center, and finally coming into contact with the style or stigma, depositing pollen on it (Roguz et al. 2018). Moreover, the arrangement of reproductive parts varies among flowers in one inflorescence. In the lower inflorescence flowers, the style is longer than the anthers, while in the flowers in the middle of the inflorescence, the style and anthers are similar in length, and the stamens touch the stigma. In the top flowers of the inflorescence, the stigma is almost half the length of the anthers and sometimes degenerates. Spontaneous self‐pollination was not recorded in *F. persica* , but it is at least partially self‐compatible (Mancuso and Peruzzi 2010).

Flowers of *F. persica* produce rather a small amount of nectar, but as a single plant produces several dozen flowers which are mostly open at the same time, the overall reward is relatively plentiful. Nectar is strongly hexose‐dominated and one flower produces, on average, 4.3 ± 4.5 μL of nectar with a sugar concentration of 46.5% ± 18.7% (Roguz et al. 2018). Nectar of *F. persica* contains a relatively small amount of AAs, with glutamine being the most abundant (Roguz et al. 2019).

### 2.2 Field Sites and Sampling

Fieldwork was conducted in 2019 on two populations located in Israel (the straightline distance between the populations is approximately 85 km; retrieved from https://earth.google.com). Plants growing in the two study populations have two different flower colors. The first field site was located in the Hakoach eucalyptus forest, near Rosh Ha'ayin (*hereafter* Rosh; 32°05′58.7″ N 34°59′04.0″ E). This population is situated at 80 m above sea level within a planted pine forest, receiving an average annual rainfall of 580 mm and an average relative humidity of 51%. The mean daily temperature is 21°C (minimum and maximum daily temperatures 16°C and 26°C, respectively). The soil is terra rossa, a red clayey soil, overlaying limestone (https://ims.gov.il/en/ClimateAtlas). Covering an area of approximately 500 m^2^, this population is estimated to consist of around 400 individual plants, with flower colors ranging from green to cream.

The second population was located in the Judean Desert, near Arad (hereafter Arad; 31°20′44.6″ N 35°07′21.8″ E). The Arad population is located at an elevation of 600 m above sea level in a desert shrubland environment with an average annual rainfall of 135 mm and an average relative humidity of 37%. The area experiences a mean daily temperature of 19°C (minimum and maximum daily temperatures 14°C and 25°C, respectively). The soil is composed of loess—fine desert dust—deposited on chalk (https://ims.gov.il/en/ClimateAtlas). Dominant vegetation includes *Artemisia herba‐alba* and *Thymelaea hirsuta*, which typify the desert shrubland ecosystem. This population spans approximately 2 km^2^ and contains an estimated 30,000 individual plants, with most flower colors being purple.

In each population, study plants were selected randomly by walking through the area and stopping at random intervals to choose a plant without prior assessment, ensuring unbiased selection. To prevent repeated sampling from the same area, plants were chosen at least 1 m apart, with sampling sites varied at each next harvest. We performed the fieldwork only in good weather conditions (no wind, no rain), between 14 and 26 February 2020 in Rosh and between 3 and 24 March 2020 in Arad, during the peak flowering time of each studied population. The nectar collection time was the same as the observations of insect visitors.

### 2.3 Nectar Collection and Analysis

We collected nectar from fritillaries in both populations to determine potential differences in nectar characteristics at a few levels: between populations (comparison based on a single flower data) and between specimens within populations (222 samples in Rosh and 104 in Arad), within inflorescence (46 inflorescences in Rosh and 33 in Arad) and a single flower (inner vs. outer whorl; 41 flowers in Rosh and 65 in Arad). In total, we collected 263 nectar samples in Rosh and 169 nectar samples in Arad. The differences in sampling effort between populations were caused by the recurring destruction of the experimental setup by the local shepherds. We selected plants during the bud stage and bagged them with nylon mesh to prevent visits by insects. We checked the progress of flowering daily in the morning and in the afternoon, and we sampled nectar during anthesis but before anther dehiscence. We collected nectar with microcapillary pipettes from nectaries of all six tepals and combined it as one sample per flower. For 43 flowers in Rosh and 50 flowers in Arad, we collected nectar separately from the outer and inner whorl to check for eventual differences. We collected nectar from the first four opened flowers in the most sampled specimens. For a few specimens, we collected nectar from all flowers in the inflorescence to check for a possible correlation between the position in the inflorescence and the nectar production. We used calibrated microcapillaries to measure nectar volume.

The collected nectar was subsequently expelled from microcapillaries into Eppendorf tubes. The samples were frozen (−20°C) until further processed. We used a refractometer prism RL‐4 (PZO, Poland) to measure nectar sugar concentration. The remaining nectar was used to assess nectar sugar and AAs composition with the use of high‐performance liquid chromatography (HPLC, methods description Supporting Information S1).

### 2.4 Breeding System

We randomly marked 64 plants in each population to study their breeding system. We marked the four lowest flowers on each plant and assigned each randomly to either of four treatments: (1) spontaneous self‐pollination—flowers were bagged with fine mesh to prevent insect visitation; (2) self‐pollination—flowers were bagged at the bud stage and we pollinated them manually with their own pollen (coming from the same flower); (3) supplemental outcross pollination—we manually transferred pollen from a donor at least 1 m away to the receptive stigma lobes; and (4) open‐pollination (control). The experimental flowers were left in the field, and we collected fruits after maturation.

### 2.5 Insect Visitation

We randomly selected 91 inflorescences in Rosh and 93 inflorescences in Arad and recorded them for 1 h using four digital cameras simultaneously. After a 1 h break, we started the next recording. Observations were conducted mainly from 9.00 am to 6.00 pm, but we also recorded flowers before sunrise and a few hours after sunset on some days. The total time of recording was 110 h during the daytime and 11.5 h before sunrise and after sunset. We analyzed the identity of flower visitors in the lab by assigning insects to morphogroups such as hoverflies, honey bees ( *Apis mellifera* ), *Eucera* (female), solitary bees (other than *Eucera*), and *Xylocopa*. Additionally, we noted the number of flowers in the inflorescence visited by each visitor and the time spent by visitors within a single flower. We also analyzed the behavior of flower visitors, whether foraging for nectar and/or pollen. Inflorescences were not excluded from subsequent rounds, which could result in the same inflorescence being observed more than once.

### 2.6 Experimental Manipulation of Access to Nectar

Access to the nectar in the outer tepals of the flowers of *F. persica* is restricted due to the inner tepals covering the large nectaries (Figure 1C). To investigate the ecological role of this feature, we conducted experiments in both study populations by uncovering the reward available in the outer whorl of tepals. We randomly selected 24 plants from each population and removed the parts of the inner tepals covering the nectaries of the outer tepals in the first four flowers. We recorded pollinator activity in experimental flowers—the time spent in one flower and inflorescence penetration (the number of flowers visited during a single visit divided by the number of flowers in the whole inflorescence). At the end of the season, we counted the number of seeds produced.

### 2.7 Statistical Analyses

We conducted data analyses using R 4.0.3 (R Core Team 2021). First, we assessed data normality of the residuals in linear model used using the Shapiro–Wilk test. Because the assumption of normality was not met, even after appropriate transformations were applied, we used the Kruskal‐Wallis ANOVA test (hereafter KW) to determine differences in nectar production and frequency of insect visitation to flowers. To investigate whether nectar sugar concentration and volume were dependent on the position of the flower within the inflorescence, we used the Kendall rank correlation (“Kendall” function in Kendall package; McLeod 2022). We analyzed the variation in amino acids composition between study populations using PERMANOVA based on Bray–Curtis distances (“adonis2” function in vegan package; Oksanen et al. 2022). Subsequently, we used the ‘envfit’ function using the NMDS ordination to identify which amino acids are driving the observed differences (vegan package; Oksanen et al. 2022). To verify that these differences were due to shifts in the location of centroids rather than differences in dispersion, we conducted an analysis of homogeneity of dispersion (“betadisper” function in vegan package; Oksanen et al. 2022). We calculated the pollen limitation index (PLI = $\frac{\text{number of seeds supplement out cross pollination}-\text{number of seeds control}}{\text{number of seeds supplement out cross pollination}}$ (number of seeds in supplement outcross pollination—number of seeds in control)/number of seeds in supplement outcross pollination) and pollinator limitation (POLI = $\frac{\text{number of fruits supplement out cross pollination}-\text{number of fruits control}}{\text{number of fruits supplement out cross pollination}}$ (number of fruits in supplement outcross pollination—number of fruits in control)/number of fruits in supplement outcross pollination; Campbell and Husband 2007). PLI was calculated as a mean value of pollen limitation index calculated for studied individual and POLI was calculated as a mean value of studied individuals.

## 3 Results

### 3.1 Nectar Production

**FIGURE 2:**
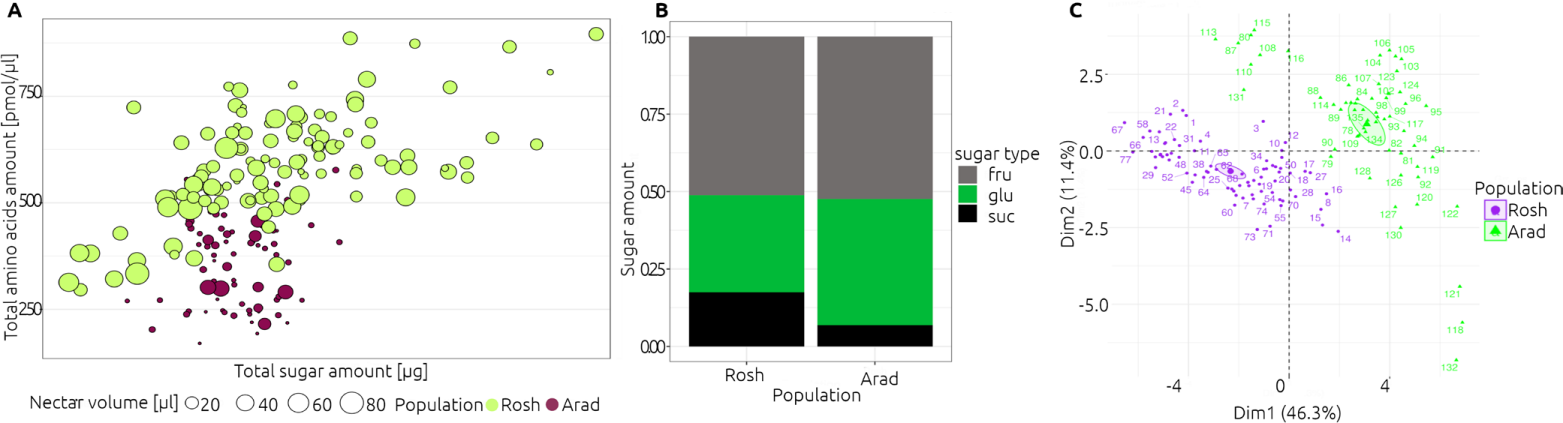
Nectar analysis in *Fritillaria persica* in studied populations (Arad, Rosh). (A) Nectar properties total sugar and amino acids amount, volume (dots scalled according to the nectar volume). (B) Relative amount of sugars expressed as the proportion of the total recorded amount within a particular population. Sugar types: Fru = fructose; glu = glucose; suc = sucrose. (C) Principal component analysis (PCA) of amino acids composition, showing the first two dimensions (PCA1‐2) of PCA that together explain 57.7% of the variance. Ellipses show concentration around each group. Individuals are labeled with a number.

The amount of nectar produced by flowers in Rosh was higher than in Arad (mean ± SE: 23.0 ± 1.16 μL and 5.65 ± 1.03 μL, respectively; KW chi‐squared = 118, df = 1, *p* < 0.01; Figure 2A, Table 1). However, the concentration of nectar did not differ between locations (20.8% ± 2.55% and 22.9% ± 0.55%, respectively; KW chi‐squared = 0.49, df = 1, *p* = 0.48; Figure 2A, Table 1). The dominant sugar in the nectar of both populations was fructose, accounting for approximately 50%, followed by glucose and sucrose, with similar proportions in both populations (Figure 2B).

**TABLE 1:** Nectar properties comparison among and within studied populations of *Fritillaria persica.* We used Kruskal–Wallis ANOVA test to determine differences in nectar production.

	Volume (μL)	Concentration (%)	Fructose amount (μg)	Glucose amount (μg)	Sucrose amount (μg)	Amino acids (pmol/μL)
*Nectar properties (among populations comparison)*						
Rosh	23.0 ± 1.16 (222)	22.9 ± 0.55 (157)	78.5 ± 1.25 (124)	48.1 ± 1.04 (124)	26.7 ± 0.40 (124)	589 ± 6.12 (124)
Arad	5.65 ± 1.03 (104)	20.8 ± 2.55 (7)	68.4 ± 0.72 (77)	40.7 ± 0.65 (77)	6.87 ± 0.27 (77)	359 ± 6.71 (77)
Test	KW rank‐sum	KW rank‐sum	KW rank‐sum	KW rank‐sum	KW rank‐sum	KW rank‐sum
*p*‐value	< 0.01	0.48	< 0.001	< 0.001	< 0.001	< 0.001

	Rosh	Arad
*Nectar properties (within populations comparison)*		
Volume inner tepal (μL)	18.8 ± 2.03 (25)	1.20 ± 0.19 (22)
Volume outer tepal (μL)	12.5 ± 2.18 (24)	2.31 ± 0.45 (22)
Test	KW rank‐sum	KW rank‐sum
*p*	0.02	0.05
Concentration inner tepal (%)	25.4 ± 1.42 (24)	NA
Concentration outertepal (%)	22.7 ± 1.36 (25)	NA
Test	KW rank‐sum	NA
*p*	0.40	NA
Amino acids inner tepal (pmol/μL)	362 ± 17.6 (26)	594 ± 14 (22)
Amino acids outer tepal (pmol/μL)	350 ± 20.6 (24)	582 ± 20.4 (24)
Test	KW rank‐sum	KW rank‐sum
*p*	0.99	0.76

	Concentration (%)		Volume (μL)	
	Rosh	Arad	Rosh	Arad
*Nectar properties versus position in the inflorescence*				
tau	0.07 (222)	0.58 (7)	−0.09 (222)	−0.05 (104)
*p*	0.19	0.11	0.06	0.47
Test	Kendall rank correlation		Kendall rank correlation	
*Tendencies in nectar properties within one plant*				
tau	−0.004	0.79	0.18	−0.02
*p*	0.94	0.03	< 0.01	0.80
Test	Kendall rank correlation		Kendall rank correlation	

In Rosh, flowers produced more nectar in the outer whorl compared to the inner (18.8 ± 2.03 μL and 12.5 ± 2.18 μL, respectively; KW chi‐squared = 5.72, df = 1, *p* = 0.02), while in Arad, flowers produced similar amounts of nectar in both the inner and outer whorls (1.20 ± 0.19 μL and 2.31 ± 0.45 μL, respectively; KW chi‐squared = 3.96, df = 1, *p* = 0.05). There were no differences in the concentration of nectar between the outer and inner whorls in Rosh (22.7% ± 1.36% and 25.4% ± 1.42%, respectively; KW chi‐squared = 0.71, df = 1, *p* = 0.40; Table 1). In Arad, the data was insufficient to draw conclusions (the low nectar production in Arad flowers limited the amount of nectar that could be sampled for sugar concentration, additionally, the sample size was constrained as the experimental setup repeatedly destroyed).

The maximum number of flowers sampled in an inflorescence was 17 in Rosh, while in Arad, it was six. Nectar sugar concentration and volume did not vary with the position of the flower within the inflorescence (from bottom to top) in Rosh (Kendall rank correlation, *t* = −0.09, *p* = 0.06 and *t* = 0.07, *p* = 0.19 for volume and concentration, retrospectively). The same was observed for Arad, where neither the volume nor the concentration of nectar showed an association with the position (*t* = 0.58, *p* = 0.11 and *t* = −0.05, *p* = 0.47). In plants from Rosh, we observed tendencies in nectar production within one inflorescence only for nectar volume (*t* = −0.18, *p* < 0.01), but not for concentration (t = −0.004, *p* = 0.94). Conversely, for plants growing in Arad, we obtained opposite results with tendencies within the inflorescence present in concentration (*t* = 0.79, *p* = 0.03) and absent for volume (*t* = −0.02, *p* = 0.80; Table 1).

We identified 26 AAs in varying proportions (Table 2). The total amount of AAs in the nectar was higher in Rosh compared to Arad (mean per flower 589 ± 118 pmol/μL and 359 ± 97.4 pmol/μL, respectively). Also, the average amount of specific AAs in all but one was higher in Rosh (*p* < 0.001, Table 2; Supporting Information S2). The principal component analysis (PCA) explained 46.3% and 11.4% of the total variation in the first two axes, respectively (Figure 2C). In Rosh, alanine was the most abundant AA, followed by glutamine and lysine, while in Arad, glutamine was the most abundant AA, followed by glycine and lysine. We did not observe differences in the total amount of AAs in nectar produced by the inner and outer whorls. The mean total amount in Rosh was 594 ± 14 pmol/μL and 582 pmol/μL in inner and outer tepals, respectively (KW chi‐squared = 0.09, df = 1, *p* = 0.76), while in Arad, it was 362 ± 17.6 pmol/μL and 350 ± 20.6 pmol/μL in inner and outer tepals, respectively (KW chi‐squared = 0.00, df = 1, *p* = 0.99; Table 1).

**TABLE 2:** Composition of nectar amino acids (pmol/μL) in *Fritillaria persica* in studied populations: Arad and Rosh; we used the Kruskal–Wallis ANOVA test to determine differences in nectar production.

Amino acid	Rosh	Arad	Chi‐squared	*p*
α‐Aminobutyric acid	6.67	3.66	24.0	< 0.001
Alanine	65.32	21.97	90.6	< 0.001
Arginine	9.35	6.35	34.3	< 0.001
Asparagine	18.58	13.01	6.15	0.013
Aspartic acid	18.58	11.01	37.1	< 0.001
β‐Aminobutyric acid	5.59	3.42	22.2	< 0.001
Citrulline	14.66	8.70	35.8	< 0.001
Cystine	17.79	13.04	34.1	< 0.001
Gamma‐aminobutyric acid	9.51	6.58	22.0	< 0.001
Glutamine	36.44	29.54	3.70	0.0545
Glutamic acid	51.75	32.31	17.9	< 0.001
Glycine	27.92	15.72	77.4	< 0.001
Histidine	18.39	11.52	22.3	< 0.001
Isoleucine	20.09	13.19	44.5	< 0.001
Leucine	48.70	23.86	84.3	< 0.001
Lysine	51.24	25.72	80.4	< 0.001
Methionine	15.09	6.87	82.7	< 0.001
Ornithine	48.90	21.98	90.9	< 0.001
Phenylalanine	11.15	7.86	40.0	< 0.001
Proline	10.97	7.41	24.9	< 0.001
Serine	23.26	28.44	13.5	0.0002
Taurine	10.53	5.82	47.0	< 0.001
Threonine	13.73	7.77	57.3	< 0.001
Tryptophan	33.19	18.23	84.9	< 0.001
Tyrosine	8.45	04.03	34.6	< 0.001
Valine	4.88	10.97	63.5	< 0.001

The results indicated a statistically significant difference in amino acids composition between study populations (PERMANOVA: *F* = 103, *R* ^2^ = 0.35, *p* < 0.001), explaining approximately 35% of the variation in the data. The test for homogeneity of multivariate dispersion was not significant (*F* = 1.7, *p* = 0.19), indicating that the observed differences in amino acids composition among populations are unlikely to be driven by differences in within‐group dispersion. These results provide strong evidence that community composition varies significantly among groups due to differences in group centroids (i.e., mean community composition), rather than unequal variability within groups. The analysis revealed that all but one amino acid differed significantly between the two studied populations, indicating distinct amino acid profiles associated with each population.

In the laboratory, nectar was diluted with water to a volume of 50 μL (10 μL of nectar +40 μL of water). The sample was filtered through spin columns using a 0.4 μm pore size membrane filter before injection. The supernatant was then loaded into the insert. An Agilent 1260 Infinity Series HPLC system with an autoinjector, refrigerated autosampler compartment, thermostatted column compartment, quaternary pump with an inline vacuum degasser, and refractive index detector was used. A ZORBAX Carbohydrate Analysis Column (4.6 mm × 250 mm, 5 μm) was used for sugar separation and analysis. A 10 μL aliquot sample or standard solution was injected. The separation was conducted at 30°C with the mobile phase comprising acetonitrile:water (70:30, v/v) at a flow rate of 1.4 mL/min. The analytical data were integrated using the Agilent OpenLab CDS ChemStation software for liquid chromatography (LC) systems. Identification of sugars was performed by comparing the retention times of individual sugars in the reference vs. test solution. The content of glucose, fructose, and sucrose was assayed based on comparisons of peak areas obtained for the samples.

Collected nectar was also analyzed for the composition of the nectar's amino acids (AAs) with the use of HPLC. After thawing the samples to an ambient temperature, the nectar was diluted to a volume of 20 μL (10 μL of nectar was mixed with 10 μL of distilled water). The sample was filtered through a spin column with a 0.4 μm pore size membrane filter (A&A Biotechnology, Poland) before injection by centrifugation for 2 min at 9000 g (relative centrifugal force). The supernatant was loaded into the insert and analyzed by an HPLC. The samples were analyzed using an Agilent Technologies 1260 Infinity series system consisting of a 1260 Infinity Agilent Quaternary pump G1311B, a 1260 Infinity Diode Array Detector (DAD) G1315D, a 1260 Infinity Fluorescence Detector (FLD) G1321B, a 1260 Infinity ALS G1329B Automated Sample Injector, a 1290 Infinity Autosampler Thermostat G1330B and a thermostatted column oven 1290 Infinity TCC G1316C. The system was controlled by Agilent OpenLab ChemStation software. The analysis of AAs in 10 μL aliquots of nectar collected from flowers was performed by gradient HPLC using an Agilent Zorbax Eclipse Plus C18 (4.6 × 150 mm, 5 μm) column with a guard, i.e., Agilent Zorbax Eclipse Plus C18 (4.6 × 12.5 mm, 5 μm). The extracts, containing primary and secondary AAs were pre‐column derivatized with o‐phtalaldehyde (OPA) and 9‐fluorenylmethyl chloroformate (FMOC) reagent. An injector program was used for the derivatization. Following derivatization, a mixture of each sample was injected into a pre‐equilibrated column operated at 40°C. The primary (OPA‐derivatized) AAs were monitored at 388 nm by DAD while the secondary (FMOC‐derivatized) AAs were monitored by FLD, at an excitation wavelength of 266 nm and an emission wavelength of 305 nm. Mobile phase A was 40 mM NaH2PO4 (pH 7.8 adjusted using 10 M NaOH solution), while mobile phase B was acetonitrile:methanol:water (45:45:10. v/v/v). The following gradient profile was seen: 0–5 min: 0% B t‐ 10% B; 5–25 min: 10% B—40.5% B; 25–30 min: 40.5% B—63% B; 30–35 min: 63% B—82% B; 35–37 min: 82% B—100 B; 37–39 min: 100% B; 39–40 min: 100% B‐ 0% B; 40 43 min: 0% B. A flow rate of 1 mL/min was used.

### 3.2 Insect Visits and Behavior

**FIGURE 3:**
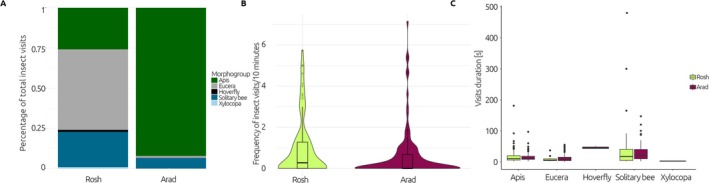
Insect visits to *Fritillaria persica* flowers in the two study populations (Arad, Rosh): (A) Expressed as the proportion of total visits for a particular population; (B) The frequency of visits (per 10 min). The frequency analysis results are summarized by boxplots, medians are given as black horizontal lines, interquartile ranges as black boxes and upper/lower adjacent values as black lines, and outliers are marked with dots. (C) The duration of visits. One outlier in solitary bees removed in Arad (480 s) and one in Rosh (300 s). The duration analysis results are summarized by boxplots, medians are given as black horizontal lines, interquartile ranges as black boxes and upper/lower adjacent values as black lines, and outliers are marked with dots.

We recorded a total of 7291 min of insect activity, with 2671 min in Rosh and 4415 min in Arad. We observed 545 insect visits to 809 flowers. In Rosh, we noted 315 visits, with most visits by *Apis mellifera* (283 visits, 89.8% of all visits), followed by solitary bees (24 visits, 7.62%) and *Eucera* bees (8 visits, 2.54%). In Arad, we recorded 230 visits, mainly by *Eucera* bees (117 visits, 50.9%), *A. mellifera* (59 visits, 25.6%), other solitary bees (49 visits, 21.3%), and few visits of *Xylocopa* and hoverflies (Figure 3A)The latter two were not recorded in Rosh. During morning visits, we observed several *Eucera* bee males sheltering overnight in the *F. persica* flowers. Bees in both populations were searching for nectar; however, some bees visited flowers solely for pollen collection. Honey bees showed a preference for freshly opened flowers with abundant pollen. Flies were observed only feeding on pollen. Although ants were seen searching for nectar in some flowers in both populations, they were excluded from the analysis due to their relatively poor pollination efficiency.

The frequency of insect visits differed among the studied populations, with 1.01 ± 0.13 visits/10 min in Rosh and 0.68 ± 0.68 in Arad (KW chi‐squared = 8.50, df = 1, *p* = 0.004; Figure 3B). There were no differences in the mean duration of visits between the studied locations (15.8 s ± 1.22 and 15.6 s ± 1.28 in Rosh and Arad, respectively; KW chi‐squared = 0.40, df = 1, *p* = 0.53). We did not find differences in the distribution of insect morphogroups in their frequency of visits among populations (Figure 3C; KW chi‐squared = 347, df = 1, *p* > 0.05). In Arad, where the population was dominated by dark morphs but with several specimens with lighter flowers, we found different frequencies of visits: 0.80 ± 0.13 visits/10 min for dark morphs and 0.09 ± 0.03 visits/10 min for light ones (KW chi‐squared = 6.72, df = 1, *p* = 0.009). All visits recorded in the light morphs were performed by *Eucera* female bees.

### 3.3 Breeding System

Measurements of fruit‐ and seed‐set were possible only in Rosh. In Arad, the plants were destroyed by local shepherds before fruit maturation. Of 129 surveyed flowers, we collected a total of 57 fruits, which contained 3523 seeds. Out of 35 supplementally pollinated flowers, 25 set fruits and produced seeds, with a mean of 63.9 ± 7.42 seeds per fruit. Only 3 out of 30 self‐pollinated flowers set fruits, with very few seeds in each (mean 2.73 ± 23.3; 27 undeveloped fruits). Only one flower out of 29 in spontaneous autogamy set a fruit (59 seeds; 28 undeveloped fruits). In control flowers, 28 out of 35 flowers set fruits, with an average of 63.7 ± 6.91 seeds. There was no difference between the number of seeds in control and supplementally pollinated flowers (*F*(3) = 0.92; *p* > 0.05). We did not detect pollen limitation, with mean PLI = −0.16 and pollinator limitation with POLI = −0.12.

### 3.4 Experimental Manipulation of Access to Nectar

**FIGURE 4:**
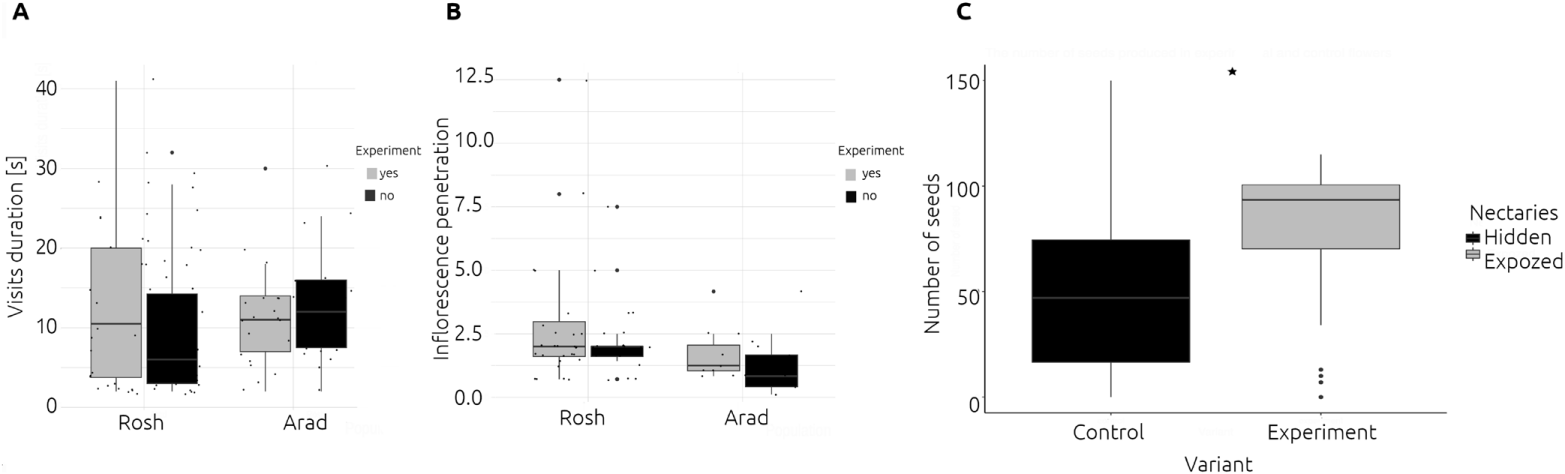
Insect visits to control and experimentally modified *Fritillaria persica* flowers in the two study populations (Arad, Rosh). (A) The duration of insects visits (s). (B) Inflorescence penetration (the number of flowers visited during a single visitd divided by the number of flowers in the whole inflorescence). The results are summarized by violin plots, medians are given as black horizontal lines, interquartile ranges as black boxes and upper/lower adjacent values as black lines, and outliers are marked with dots. (C) The seed production from control and experimentally modified *F. persica* flowers in Rosh differed significantly (Kruskal–Wallis *W* = 11.3; *p* < 0.001). The seed production analysis results are summarized by boxplots, medians are given as black horizontal lines, interquartile ranges as black boxes and upper/lower adjacent values as black lines, and outliers are marked with dots.

During the course of the project, we recorded 72 visiting insects, which overall spent 2021 min on experimental flowers (1124 min in Rosh 897 and minutes in Arad). Only *A. mellifera* were recorded on the experimental plants in Rosh, while only *Eucera* bees (female) were observed in Arad. There were no differences in the duration of visits between experimental and control flowers in the studied locations. In Rosh, the mean duration in experimental and control flowers was 15.5 ± 1.15 and 12.8 ± 1.66, respectively (KW chi‐squared = 0.32, df = 1, *p* = 0.57), while in Arad, the mean duration in experimental and control flowers was 10.8 ± 1.24 and 16.0 ± 1.30, respectively (KW chi‐squared = 0.10, df = 1, *p* = 0.75; Figure 4A). In both populations, the inflorescence penetration (the number of flowers visited during a single visit divided by the number of flowers in the inflorescence) was higher in experimental flowers (Figure 4B). In Rosh, the mean penetration in experimental and control flowers was 2.75 ± 0.46 and 0.23 ± 0.04, respectively (KW chi‐squared = 69.2, df = 1, *p* < 0.01), while in Arad, the mean penetration in experimental and control flowers was 1.65 ± 0.28 and 0.15 ± 0.02, respectively (KW chi‐squared = 33.1, df = 1, *p* < 0.01). We collected all fruits from the 46 experimental flowers, where we found 3684 seeds. Out of 46 experimental flowers, 44 produced seeds (two fruits not found), with a mean of 80.1 ± 4.71 seeds. The number of seeds produced in experimental flowers was higher than in control flowers (51.0 ± 41.6, KW chi‐squared = 11.3; df = 1, *p* < 0.001, Figure 4C).

## 4 Discussion

Adaptation to pollinator preferences is often described as a driving force for the evolution of floral trait variation, particularly regarding nectar properties. This study explores the differences in the reward properties of two color morphs of *F. persica* and their interaction with pollinators. Nectar sugar concentration and profile were generally constant in studied populations; however, we recorded differences only in nectar volume and AAs concentration. These results suggest that the observed variation in nectar production may be caused by environmental factors, rather than genetic differences between plants. The differences between Rosh and Arad are not a consequence of local adaptation to different pollinator communities, as they remained qualitatively similar, with all main morphogroups present in both study populations, although their contribution was variable. In interpreting these findings, it is essential to recognize the limitations associated with our study's small sample size and the absence of population replication. The observed variation in nectar production and the consistency in visitor assemblages across populations provide valuable insights, but the restricted sample size limits the generalizability of the obtained results. Moreover, as we did not use a common garden or experiments disentangling plastic from genetic differences between populations, we can not exclude genetic differences existing as a consequence of random drift or adaptation to environmental factors.

By manipulating flower parts, we demonstrated that uncovering the hidden nectaries does not influence the time spent in the flower; however, in manipulated inflorescences, pollinators visited more flowers. Thus, by studying the reward properties and plant–pollinator interaction of the two color morphs, we can infer some of the drivers of the flower features evolution in *F. persica* .

### 4.1 Nectar Properties

Our study shows that *F. persica* flowers produce hexose‐rich nectar, consistent with a previous study conducted in cultivation. This suggested that *F. persica* is an insect‐pollinated species producing nectar dominated by hexose sugars (Roguz et al. 2018). However, in wild populations, we observed a higher nectar volume (up to an order of magnitude in Rosh), coupled with a much lower sugar concentration, while the nectar sugar composition remained similar to the previous study (Roguz et al. 2018).

Glutamine, alanine, glycine, and lysine were the most abundant AAs found in the studied populations, with the former two being among the most prevalent AAs found in nectar (Baker and Baker 1975; Erhardt and Rusterholz 1998). Glycine and lysine are also recognized as promoting insect growth (Dadd 1973). Surprisingly, we observed low quantities of proline, despite its common occurrence in the nectar of many plant species and its particular importance for Hymenoptera pollinators, as it facilitates rapid ATP production and fuels the initial phase of flight (Carter et al. 2006; Micheu et al. 2000). The presence of small amounts or absence of proline is not uncommon and has been found in other *Fritillaria* species as well as in other eudicot insect‐pollinated species, such as *Polemonium caeruleum* (Ryniewicz et al. 2020).

Glutamine was found to be dominant in the nectar of *F. persica* plants from Arad. However, the amount of glutamine detected in the wild populations was an order of magnitude lower than that reported by Roguz et al. (2021). Despite detecting a higher number of AAs in the wild populations compared to the previous study, we observed a different AAs composition and the absence of certain AAs, including citrulline, arginine, β‐alanine, taurine, gamma‐aminobutyric acid, β‐aminobutyric acid, α‐aminobutyric acid, methionine, norvaline, leucine, and proline. Variations in nectar characteristics have been previously reported in other species, such as *Impatiens capensis* (Lanza et al. 1995), and may result from large‐scale geographic differentiation or cultivation under garden conditions. Factors such as temperature, available sunlight, available water, and the presence or absence of developing fruit can cause variations in nectar characteristics (Lanza et al. 1995).

We observed a higher amount of nectar production in the hidden nectaries of the outer tepals only in Rosh, suggesting geographic variations in nectar production between the outer (hidden) and inner (accessible) nectaries. These observed differences may reflect variations in the distribution of nectar‐producing cells within the flower, with the outer tepals potentially harboring a higher density of nectar‐producing cells.

We found almost no differences in nectar volume and concentration among individual plants within each population, which was unexpected, given that studies on other species have often shown significant variations in nectar production between individuals (e.g., Lanza et al. 1983; Real and Rathcke 1991). The homogeneity in nectar production is uncommon among plants since nectar properties, as a physiological trait, are influenced by plant condition and habitat properties (Brown et al. 2012; Cruden 1977; Gijbels et al. 2014; Lanza et al. 1995; Ryniewicz et al. 2020). For instance, studies on *Asclepias* have revealed that nectar production is related to physiological traits such as root weight and the number of flowers in the inflorescence (Pleasants and Chaplin 1983), while studies on *Kalmia latifolia* have shown that nectar production is associated with visitation frequency (Real and Rathcke 1991). The absence of differences in nectar volume and concentration at the individual level in our study suggests that the observed among‐population variation in nectar production is likely to be driven by environmental factors; however, genetic differences between different color morphs can not be excluded.

Other nectar characteristics in studied species, namely sugar concentration and sugar profile, remained consistent within populations in both sites. The differences between populations can be attributed to differences in environmental factors—desert plants produced less nectar, consistent with the idea that desert plants may face a range of challenges that limit their ability to produce nectar, like water availability, high temperatures, and intense sunlight (Lanza et al. 1995). Furthermore, the consistent nectar concentration between desert plants and those from other environments suggests that desert plants might allocate their limited resources towards producing high‐quality nectar rather than increasing its quantity. This strategic allocation could ensure that the nectar produced by desert plants remains attractive to pollinators as an energy source, even when available in lower quantities. Although populations vary in the extent of flower color variation, we cannot draw conclusions regarding the relationship between flower color and variations in nectar production within flowers, as these factors may be confounding.

Our results confirm the conservative nature of nectar composition at the species level, consistent with previous findings (Baker and Baker 1986). While the AAs profile was similar across the studied populations, notable differences were observed. Specifically, the average amount of AAs and the quantity of almost all AAs in the nectar were higher in the Rosh population compared to Arad, with variations in the most abundant AAs between populations. These differences could be due to a range of environmental factors, such as soil nutrients, especially nitrogen, which are required for AA synthesis in plants (Brzosko et al. 2021; Ryniewicz et al. 2020). The differing nitrogen supply in the studied populations, experiencing contrasting environments (planted Mediterranean forest vs. desert), likely contributed to the observed differences in AAs composition. The relatively small differences in AAs profiles between populations suggest that AAs concentration and composition may not be primarily under pollinator‐mediated selection.

This similarity may indicate that, in the context of plant–pollinator interactions, *F. persica* adopts a relatively generalist strategy, potentially attracting a broad range of pollinators. However, this strategy is not fully realized due to limitations in the local entomofauna, which restrict the diversity and abundance of available pollinators in each population. Pollinator‐mediated selection is weaker when the primary visitors are multiple animal taxa (Caruso et al. 2019). For species visited and pollinated by a wide spectrum of animals, spatial differences in nectar characteristics will not result in differences in pollinator assemblages and local specialization. For example, in the case of the generalist *Angelica sylvestris* , where different populations exhibit varying nectar and scent profiles and are effectively pollinated by disparate pollinator morphogroups, transplantation experiments revealed that reproductive success was not related to the source of experimental plants and that the insects do not exhibit preferences toward local genotypes (Roguz et al. 2019).

### 4.2 Breeding System

We did not detect any pollen limitation, as the reproductive success of *F. persica* was nearly identical between hand‐ and open‐pollinated flowers. Spontaneous autogamy occurs very rarely, if at all, in the lower flower. We need further studies testing spontaneous autogamy in the middle flowers with style and anthers at the same height. Upper flowers were mostly males, obviously unable to produce seeds. Overall, our results indicate the presence of some level of self‐incompatibility in *F. persica* . The low ability to self‐pollinate in most bottom flowers in *F. persica* may be related to pollinators' behavior. In a vertical inflorescence that is visited by bees that tend to forage upwards, the lowermost flowers receive outcross pollen while the uppermost flowers export it to other plants (Barrett 2003). Additionally, in cases of sufficient transfer of pollen grains, securing maximal reproductive success, there is no strong selective pressure for maintaining self‐compatibility. However, in the case of recurring mismatch with pollinators' activity, some self‐compatible individuals may secure seed production.

### 4.3 Interaction With Pollinators

Flowers in the Arad population were predominantly visited by wild bees, mostly *Eucera*, whereas flowers in the Rosh population were almost exclusively visited by honey bees. The presence of cultivated honey bee colonies may have influenced the pollination system in *F. persica* and local plant–pollinator interactions. A study on *Brassica rapa* has demonstrated that changes in pollinator communities can have rapid consequences on the evolution of plant traits and mating systems (Gervasi and Schiestl 2017). While we lack direct evidence for such changes in *F. persica* , the perennial growth form of the species suggests that any effects of modified pollination communities may have been gradual and facilitated by interactions with environmental conditions. Nonetheless, the effect of a cultivated honey bee on plant reproductive success in the Mediterranean climate region in Israel has been demonstrated for other ornamental geophytes, such as *Iris atropurpurea* (Watts et al. 2013). Honey bee overflow in natural communities may increase pollen limitation and saturate the opportunity for selection (Trunschke et al. 2017). This can also explain the difference in nectar characteristics in Rosh, compared to the Arad population.

In desert populations, dominated by individuals with dark flowers, we also record night‐sheltering pollinators entering flowers shortly before dusk. The dark coloration may be related to gathering heat by absorbing solar radiation. This thermal reward was previously described in flowers offering also a food reward (Herrera 1995; Totland 1996). Contrary to *Oncocyclus* irises, another desert species where sheltering male solitary bees have been described as obligatory pollinators (Sapir et al. 2005, 2006), *F. persica* does not depend on night‐sheltering male bees as its obligatory pollinators, and their role in the pollination of this species may be marginal. We recorded several *Eucera* specimens grouped tightly together in flowers mostly at the end of the flowering period, with these flowers typically lacking pollen grains in the anthers.

The Arad population is predominantly composed of dark‐colored flowers, with a lower frequency (approximately 5% of the population) of light‐colored flowers. We have recorded a lower visitation frequency for the light‐colored flowers. We assume that, in the case of the studied species, flower color may not be an adaptive feature, and color polymorphism is not the outcome of an adaptive process. The observed within‐population color variation may be instead a result of neutral or random processes (Sapir et al. 2021), as in the case of *Iris lutescens* , where studies testing genetic causes of color dimorphism revealed that the relative frequency of yellow versus purple flowers within populations is affected by drift or gene flow, rather than ecological factors (Wang et al. 2014).

Color polymorphis in this species or population may also cause morph‐specific combinations of floral traits that attract pollinators with different preferences, e.g., towards flower scent. In some cases, white flower morphs might release more aromatic scent compounds because they represent null mutants with blocked biosynthetic pigment pathways, which can lead to changes in the type or amount of volatile compounds produced (Majetic et al. 2007). The potential for biotic agents such as pollinators to select for particular floral scent–color combinations needs further studies in the case of *F. perisca*.

Flower color in *F. persica* may serve as a phenotypic filter, influencing interactions with both pollinators and herbivores. Research indicates that flies, particularly hoverflies like *Eristalis tenax* , prefer yellow and white flowers over purple ones. This preference is likely due to their visual sensitivity to yellow wavelengths, which makes yellow flowers more visible and attractive. Furthermore, in terms of herbivore interactions, *Raphanus sativus* red morphs, which are richer in secondary metabolites, offer better defense against herbivores (Irwin et al. 2003).

Pollinator‐dependent flowers rely on imported carbohydrates from surrounding leaves for reward production. Floral photosynthesis in green petals may also contribute to carbon requirements for reproduction (Aschan and Pfanz 2003). Beyond pollination, differences in flower color in *F. persica* may be linked to pigment accumulation, such as flavonoids and anthocyanins, which can protect against photooxidative stress, cold, and water stress (Chalker‐Scott 1999). This protection is particularly advantageous in environments with high solar radiation, such as deserts, improving flower longevity and nectar quality.

Further studies focusing on the efficiency of flower visitors are needed to fully describe the pollination ecotypes. The potential selective pressure exerted by variable pollinators is a necessary precondition for the local specialization of the most important pollinators (Gómez and Zamora 2006; Zamora 2000; Zych et al. 2019).

### 4.4 Experimental Flower Manipulation

*Fritillaria persica* flowers have partly hidden nectar, and our studies revealed that the amount of hidden nectar may exceed that of the visible fraction—in the Rosh population, the outer tepals produced more nectar. Recording of pollinators' behavior within the flowers showed that bees, such as honeybees, were able to detect the reward from hidden nectaries. Their visits were similar in duration between control and experimental flowers, where the nectaries were uncovered. However, differences were observed in the number of flowers visited during a single bout—in both studied populations, pollinators visited more flowers within the inflorescences when the nectaries of the bottom flowers were uncovered.

Taking into consideration a higher number of flowers visited within one inflorescence, the recorded higher reproductive success of flowers with uncovered nectaries, when compared to control flowers, may be surprising. We hypothesize that this positive effect on seed production may be related to potential changes in the behavior of pollinators in experimentally modified flowers. In flowers with all nectaries available, pollinators may move less while foraging (as access to the reward is easier) thereby depositing fewer of their own pollen grains on the stigma. In partially self‐incompatible species, which probably is the case of *F. persica* , visiting more flowers within one inflorescence or depositing more of their own pollen while foraging may result in a high level of geitonogamy and reduced reproductive success (Webb and Lloyd 1986). Therefore, the partially hidden reward may discourage flower visitors from visiting more flowers within one inflorescence.

In the ecological context, the presence of hidden nectaries may function as a strategy to protect food rewards from nectar robbers, consuming nectar without providing any pollination services to the plant, such as ants, which were observed in the flowers of the studied species. Additionally, we assume that the role of hidden rewards in the case of *F. persica* may also be maintaining the attractiveness of flowers over a longer time. As the amount of nectar in the outer hidden tepals accumulates during anthesis, it gradually pours into the inner visible nectaries, extending the period of attractiveness for pollinators and increasing the likelihood of more pollinator visits.

## 5 Conclusions

Determining the drivers of spatial variation in natural populations is crucial to understanding the process of adaptive differentiation and the diversity of flowering plants. Our study documented geographical variation in the plant–pollinator interactions of two populations, differing in their flower colors. This suggests that the surveyed populations may represent distinct pollination ecotypes. The visitor assemblages recorded in both populations remained similar qualitatively, with all main morphogroups present in both study populations, but their contribution was variable. Reward properties were also similar among studied populations—nectar concentration and composition were almost the same among studied populations. This similarity may indicate that in the context of plant–pollinator interactions, *F. persica* represents a rather generalistic strategy. The reward, although partly hidden, is available for pollinators and may extend the attractiveness of flowers to potential pollinators. By manipulating the flower parts, we show that uncovering the hidden nectaries does not influence the time spent in the flower; however, in manipulated inflorescences, pollinators visited more flowers. Overall, our observations highlight the importance of studying plant–pollinator interactions in detail, as they can reveal important and previously unknown aspects of plant biology and ecology. Further studies testing the selective pressure exerted by local assemblages of pollinators are needed to reveal the potential directions of species evolution.

## Author Contributions

**Katarzyna Roguz:** conceptualization (lead), data curation (lead), formal analysis (lead), funding acquisition (lead), investigation (lead), methodology (lead), project administration (lead), validation (lead), writing – original draft (lead), writing – review and editing (lead). **Paweł Pstrokoński:** data curation (supporting), investigation (supporting), writing – review and editing (supporting). **Justyna Ryniewicz:** data curation (supporting), investigation (supporting), writing – review and editing (supporting). **Magdalena Chmur:** investigation (supporting). **Andrzej Bajguz:** investigation (supporting), writing – review and editing (supporting). **Yuval Sapir:** conceptualization (supporting), methodology (supporting), writing – original draft (supporting), writing – review and editing (supporting).

## Conflicts of Interest

The authors declare no conflicts of interest.

## Supporting information


**Supporting Information S1.** Description of materials and methods used for nectar sugar and aminoacids concentration and composition.

**Supporting Information S2.** The amount of specific amino acids (pmol/μL) in studied populations. Thick lines are medians, boxes are interquartile ranges (25th and 75th percentile; box edges), and 10th and 90th percentile (whiskers) of the calculated data. The significance of difference between the number of own and heterospecific pollen grains was analyzed by the Wilcoxon signed‐ranks test. Variants with statistically significant differences (statistical significance determined at *p* = 0.05) marked with an asterisk.

## Data Availability

All data used in the study are available at https://zenodo.org/records/12658287?token=eyJhbGciOiJIUzUxMiJ9.eyJpZCI6ImU5NTg0ZGQxLTRhNWMtNDVkYy05YWQ3LWY1NzcxYjg4MTMxNiIsImRhdGEiOnt9LCJyYW5kb20iOiIyNzJhMTE1OWY5MTNkYTYzNzJhYzQ5ZjIwZTZmYjMwZCJ9.__Ve2NHFDBD_qX6lRHXNeXUOQsOx4MGUVpbRvubP2uANP2Ho_frBoivGi0aSSAXKCdAAy500u8mWqLhrnAZW5A.
