# Supplementary material for: Environment or Pollinators? Factors Shaping Breeding System and Spatial Variation in Nectar Properties and Pollination System in a Desert Species Fritillaria persica L. (Liliaceae)
Source: Ecol Evol. 2025 Apr 25;15(4):e71265. doi: 10.1002/ece3.71265 (PMC12022800; doi:10.1002/ece3.71265)
Supplement: Supplementary file 1 — Supporting Information S1. Description of materials and methods used for nectar sugar and aminoacids concentration and composition. [file ECE3-15-e71265-s002.docx]

In the laboratory, nectar was diluted with water to a volume of 50 µl (10 µl of nectar + 40 µl of water). The sample was filtered through spin columns using a 0.4 µm pore size membrane filter before injection. The supernatant was then loaded into the insert. An Agilent 1260 Infinity Series HPLC system with an autoinjector, refrigerated autosampler compartment, thermostatted column compartment, quaternary pump with an inline vacuum degasser, and refractive index detector was used. A ZORBAX Carbohydrate Analysis Column (4.6 mm× 250 mm, 5 µm) was used for sugar separation and analysis. A 10 µl aliquot sample or standard solution was injected. The separation was conducted at 30◦C with the mobile phase comprising acetonitrile:water (70:30, v/v) at a flow rate of 1.4 ml/min. The analytical data were integrated using the Agilent OpenLab CDS ChemStation software for liquid chromatography (LC) systems. Identification of sugars was performed by comparing the retention times of individual sugars in the reference vs. test solution. The content of glucose, fructose, and sucrose was assayed based on comparisons of peak areas obtained for the samples.

Collected nectar was also analyzed for the composition of the nectar’s amino acids (AAs) with the use of HPLC. After thawing the samples to an ambient temperature, the nectar was diluted to a volume of 20 μL (10 μL of nectar was mixed with 10 μL of distilled water). The sample was filtered through a spin column with a 0.4 µm pore size membrane filter (A&A Biotechnology, Poland) before injection by centrifugation for 2 min at 9000 g (relative centrifugal force). The supernatant was loaded into the insert and analyzed by an HPLC. The samples were analyzed using an Agilent Technologies 1260 Infinity series system consisting of a 1260 Infinity Agilent Quaternary pump G1311B, a 1260 Infinity Diode Array Detector (DAD) G1315D, a 1260 Infinity Fluorescence Detector (FLD) G1321B, a 1260 Infinity ALS G1329B Automated Sample Injector, a 1290 Infinity Autosampler Thermostat G1330B and a thermostatted column oven 1290 Infinity TCC G1316C. The system was controlled by Agilent OpenLab ChemStation software. The analysis of AAs in 10 μL aliquots of nectar collected from flowers was performed by gradient HPLC using an Agilent Zorbax Eclipse Plus C18 (4.6 × 150 mm, 5 μm) column with a guard, i.e. Agilent Zorbax Eclipse Plus C18 (4.6 × 12.5 mm, 5 μm). The extracts, containing primary and secondary AAs were pre-column derivatized with o-phtalaldehyde (OPA) and 9-fluorenylmethyl chloroformate (FMOC) reagent. An injector program was used for the derivatization. Following derivatization, a mixture of each sample was injected into a pre-equilibrated column operated at 40 °C. The primary (OPA-derivatized) AAs were monitored at 388 nm by DAD while the secondary (FMOC-derivatized) AAs were monitored by FLD, at an excitation wavelength of 266 nm and an emission wavelength of 305 nm. Mobile phase A was 40 mM NaH2PO4 (pH 7.8 adjusted using 10 M NaOH solution), while mobile phase B was acetonitrile:methanol:water (45:45:10. v/v/v). The following gradient profile was seen: 0–5 min: 0% B t- 10% B; 5- 25 min: 10% B - 40.5% B; 25–30 min: 40.5% B - 63% B; 30–35 min: 63% B - 82% B; 35–37 min: 82% B - 100 B; 37–39 min: 100% B; 39–40 min: 100% B- 0% B; 40 43 min: 0% B. A flow rate of 1 mL/min was used.
