# Supplementary figures and images for: Environment or Pollinators? Factors Shaping Breeding System and Spatial Variation in Nectar Properties and Pollination System in a Desert Species Fritillaria persica L. (Liliaceae)
Source: Ecol Evol. 2025 Apr 25;15(4):e71265. doi: 10.1002/ece3.71265 (PMC12022800; doi:10.1002/ece3.71265)

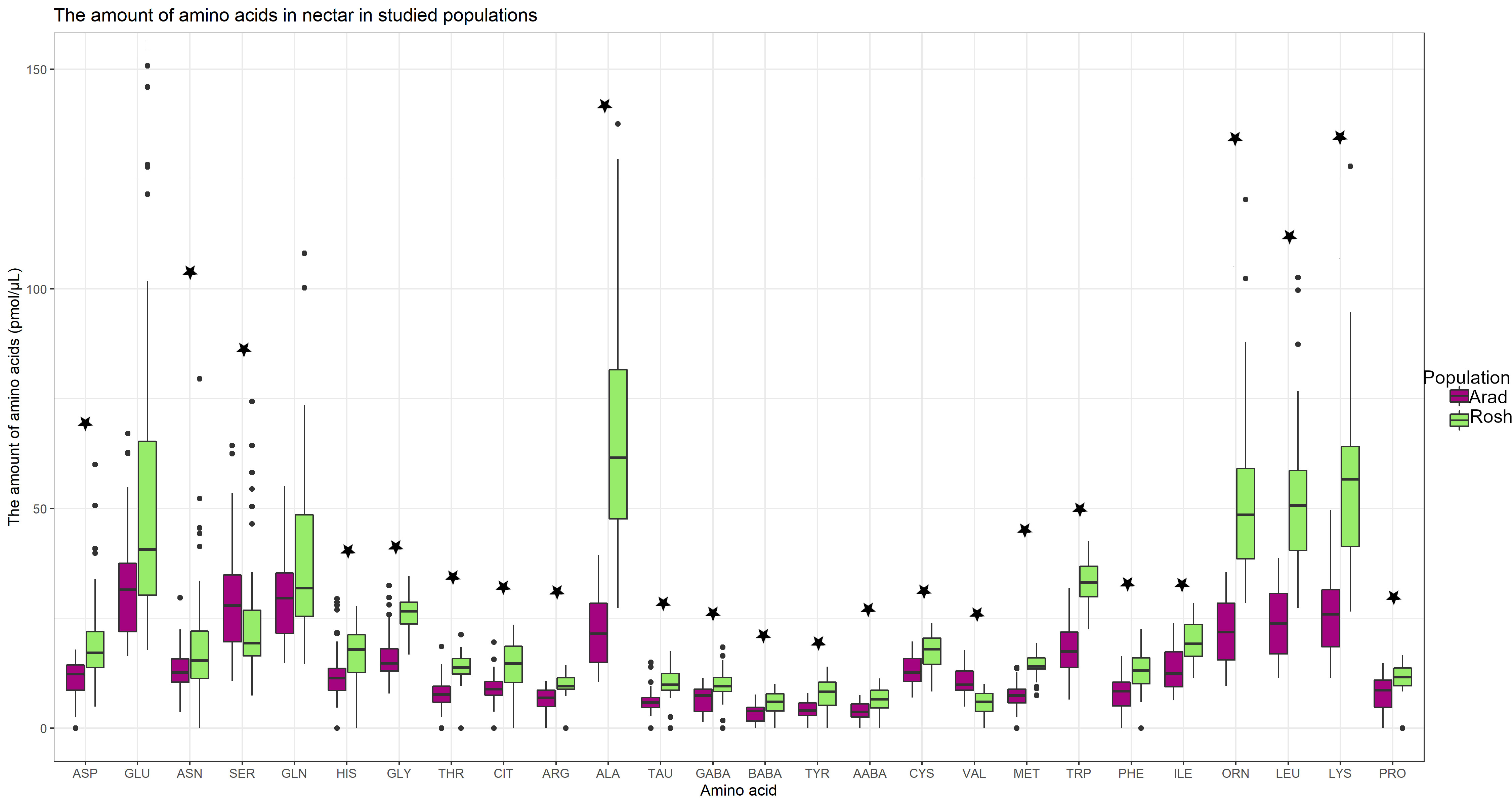

Supplement: Supplementary file 2 — Supporting Information S2. The amount of specific amino acids (pmol/μL) in studied populations. Thick lines are medians, boxes are interquartile ranges (25th and 75th percentile; box edges), and 10th and 90th percentile (whiskers) of the calculated data. The significance of difference between the number of own and heterospecific pollen grains was analyzed by the Wilcoxon signed‐ranks test. Variants with statistically significant differences (statistical significance determined at p = 0.05) marked with an asterisk. [file ECE3-15-e71265-s001.jpg]
